# Deciphering Photoluminescence in an Aryl Iodides–Gold Nanoparticles System: Au-Mediated Homocoupling Reaction at a Low Temperature

**DOI:** 10.1021/acs.jpclett.4c00346

**Published:** 2024-04-04

**Authors:** Paulina Rajchel-Mieldzioć, Piotr Fita

**Affiliations:** Institute of Experimental Physics, Faculty of Physics, University of Warsaw, Pasteura 5, 02-093 Warsaw, Poland

## Abstract

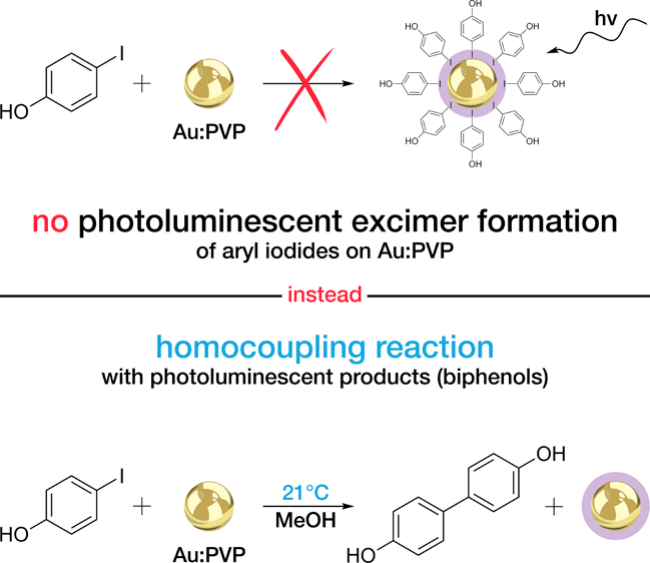

The study of photoactive materials often unveils intriguing findings, showcasing the value of an interdisciplinary approach. We examined the purported metal-enhanced luminescence thought to result from the chemisorption of aryl iodides on poly(*N*-vinylpyrrolidone)-stabilized gold nanoparticles. Our discovery deviates from previous assumptions: the fluorescence observed does not originate from excimers of iodophenols chemisorbed on Au:PVP. Instead, it arises from biphenol products, resulting from a gold-mediated Ullmann homocoupling reaction that occurs within the system. Notably, this reaction, known for its demanding nature, proceeds in methanol under purely ambient conditions: room temperature and air atmosphere, without the need for a base. Therefore, these findings not only offer a complete understanding of the observed luminescence but also provide a substantial contribution to the field of carbon–carbon coupling reactions.

The search for new photoactive materials and analysis of their photophysical properties are of utmost importance as a result of the variety of applications of photoactive systems. Of particular note are photoactive materials based on metallic nanoparticles, including colloidal nanomaterials capable of avalanche photon emission,^1^ systems exhibiting metal-enhanced fluorescence,^2^ or emitters for organic light-emitting diodes.^3^ Such complex materials can have high fluorescence yields and allow for the implementation of modifications to enhance their biocompatibility. For this reason, significant effort is currently put into development of nanomaterials functionalized with organic ligands.^4,5^

Although the search for highly efficient photoactive systems is an exciting pursuit, it is crucial to exhibit caution and avoid misinterpretation of experimental results, which may lead to unwarranted conclusions regarding the discovery of novel systems. When organometallic nanomaterials are synthesized, it is important to consider the catalytic properties that metallic nanoparticles exhibit in various chemical reactions to take into account the occurrence of any possible side processes.

The paper of Maity et al.^6^ described a unique photophysical phenomenon associated with aryl iodides chemisorbed on gold nanoparticles. Namely, the authors observed that a mixture of 4-iodophenol with gold nanoparticles in methanol exhibited strong fluorescence after ultraviolet (UV) excitation. Undoubtedly, this observation was quite astonishing because 4-iodophenol is a poor fluorophore itself, and gold nanoparticles are known to quench fluorescence. Thus, formation of the fluorescent adduct would be an unexpected effect with significant consequences for the field of organometallic photoactive materials. The occurrence of a chemical reaction was not considered; instead, the authors concluded that the observed fluorescence originates from excimers of aryl iodides chemisorbed on Au:PVP. This proposed solution was heuristic, but after our investigation, the reality turned out to be even more astonishing.

In this work, we report that a simple mixture of aryl iodides with Au:PVP in MeOH does not result in chemisorption and excimer formation, as previously reported, but instead leads to an Ullmann homocoupling reaction under remarkably mild conditions.

Successful attempts have already been made to carry out the said reaction (synthesis of biaryls) using gold as a mediator in the recent past.^7−9^ However, it is worth noting that the conditions for this reaction can be quite demanding, which, to our delight, is not the case in the presented work. For the experiments, PVP-stabilized Au:PVP nanoparticles (less than 2 nm in diameter; Figure S1 of the Supporting Information) were synthesized according to the method previously described in the literature.^10^ The resulting solutions were subjected to centrifugal ultrafiltration, washed, and then lyophilized for further use in MeOH solutions (details of the experimental procedures are given in the Supporting Information).

Upon dropping a methanolic solution of the substrate, 4-iodophenol or 2-iodophenol, into a solution of Au:PVP in MeOH, the appearance of a relatively strong emission red shifted with respect to the weak fluorescence of corresponding iodophenol was immediately observed, exactly as reported in ref (6). A careful analysis of the fluorescence spectra of the solutions allowed for solving the mystery of this emission: the spectra were typical of the products of the coupling reaction, 4,4′-biphenol and 2,2′-biphenol, respectively.^11^ Most significantly, however, the observed fluorescence is exhibited not only by the solution in the presence of Au:PVP nanoparticles but also and above all in the supernatant collected after Au:PVP centrifugation, which contains only negligible amounts of gold, as confirmed by inductively coupled plasma mass spectrometry (ICP MS) analysis included in the Supporting Information.

The latter fact confirms that we are not dealing with a fluorescent material based on metallic nanoparticles but with the products of the reaction mediated by them. This is also indisputably evidenced by transmission electron microscopy (TEM) analysis combined with energy-dispersive X-ray (EDX) mapping. As shown in Figure 1, the nanoparticles after the reaction have agglomerated to some extent and are covered with iodine, but in the supernatant, which shows emission spectra specific to biphenols, the presence of iodine and gold is negligible.

**Figure 1:**
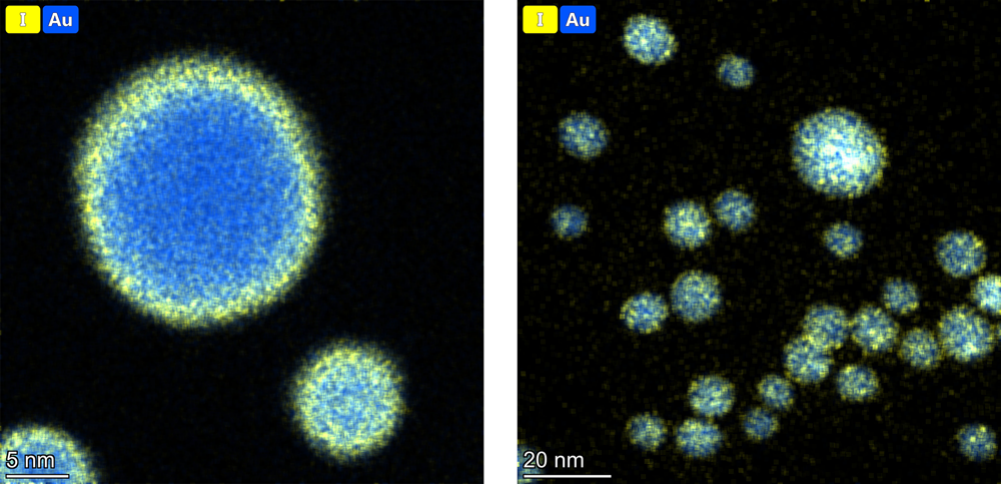
TEM images of Au:PVP centrifuged after the reaction with 4-iodophenol. Colors are applied according to EDX mapping results: blue, gold; yellow, iodine.

The conclusions from the combined results of the experiments mentioned above are the following: The fluorescent species formed in the studied system contain neither gold nor iodine. Instead, their fluorescence spectra correspond to the spectra of products of the coupling reaction of iodophenols used in the study. Iodine, in turn, remains adsorbed at the surface of the gold nanoparticles. Thus, we are witnessing the Ullman homocoupling reaction, and what is indeed notable is that the material used (Au:PVP) is characterized by a low degree of complexity and allows the reaction to be carried out under ambient conditions of room temperature (21 °C) and without the addition of a base.

The aim of this work was not to optimize the ongoing process but to describe it in contrast to the earlier proposed interpretation based on the assumption of metal-enhanced luminescence by chemisorbed aryl iodides on gold particles. Through an examination of the photophysical properties of both the substrates and products, it was revealed that it is feasible to accomplish this task without reliance on nuclear magnetic resonance (NMR) spectroscopy. However, to provide irrefutable confirmation, we included NMR analysis in the Supporting Information. This analysis, confirming the presence of biphenols, complements the primary reasoning based on optical spectroscopy, thereby strengthening our overall findings. As stated above, the reaction products appear immediately after mixing the substrates with Au:PVP, but the conversion continues for some time. Thus, the resulting products were separated from gold nanoparticles for a more in-depth spectroscopic analysis by centrifugation 24 h after the initiation of the reaction. In the meantime, the solution was not subjected to heating or stirring, and no reactants were added. The process was carried out for five sets of initial concentrations of substrates and the gold nanoparticles (AuNPs), for both 2-iodophenol and 4-iodophenol; details are shown in Table 1.

**Table 1:** Substrates, Products, and Estimated Yields of the Ullman Homocupling Reactions Mediated by Au:PVP (1 Atomic %) in MeOH

entry	Au:PVP^a^ (μM)	iodophenol^b^ (μM)	yield (%) of 4,4′-biphenol	yield (%) of 2,2′-biphenol
1	100	30	97	68
2	50	30	35	40
3	200	30	87	85
4	100	15	73	87
5	100	60	32	31

a Concentration of Au:PVP in relation to atomic gold, described in detail in the Supporting Information

b Concentration of the substrate, 4-iodophenol or 2-iodophenol, respectively.

For comparison to the reaction products, the fluorescence spectra of commercially available biphenols in neat MeOH solutions have been recorded. The fluorescence spectrum of pure 4,4′-biphenol (Figure S6 of the Supporting Information) is dominated by the band corresponding to its neutral form (present in MeOH solution, with the excitation maximum at 275 nm and the emission maximum at approximately 353 nm). It is also possible to detect fluorescence of its anionic (deprotonated) form in the alkalized solution (weak fluorescence, with the excitation maximum at 293 nm and the emission maximum at approximately 420 nm as a result of the mixture of deprotonated forms). The emission spectrum of the 4-iodophenol coupling reaction product showed exceptional agreement with that of pure 4,4′-biphenol (panels a and b of Figure 2), which proves high selectivity toward this isomer. Moreover, the obtained fluorescence decay completely coincides with the fluorescence decay of standard 4,4′-biphenol (monoexponential, with the decay time of 7.1 ns; Figure 2c). Thus, the yield of the coupling reaction could be accurately determined by the comparison of the fluorescence intensity of the post-reaction supernatant and the standard solution (Figure 2a; details are provided in the Supporting Information). The reaction yield reached a high value of 97% for the most optimal set of initial concentrations (entry 1 in Table 1).

**Figure 2:**
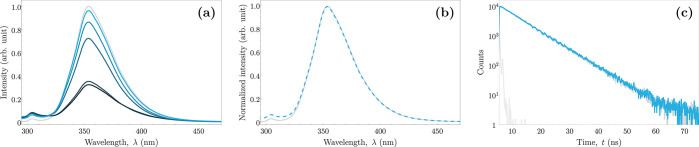
Emission spectra and fluorescence decays of the product obtained by coupling of 4-iodophenol (blue) and reference solution of 4,4′-biphenol (gray). (a) Comparison of the fluorescence spectra of the 5 μM 4,4′-biphenol (standard) solution and the obtained product for each set of initial concentrations recorded with excitation at 275 nm; a lighter shade of blue corresponds to a higher yield (1 > 3 > 4 > 2 > 5; Table 1). (b) Normalized emission spectra of the standard solution and the coupling product (for set 1). The short-wavelength edge of the spectrum of the post-reaction solution shows a subtle contribution from unreacted 4-iodophenol. (c) Fluorescence decays recorded at 353 nm with excitation at 275 nm. The light gray color is the instrument response function (IRF).

The fluorescence spectrum of pure 2,2′-biphenol (Figure S7 of the Supporting Information) depends upon its protonation state. For the neutral form (present in MeOH solution), the emission maximum is observed at approximately 347 nm (excitation maximum at approximately 286 nm). The monoanion (the deprotonated form, predominant in the alkalized MeOH solution), in turn, shows strong fluorescence with an emission maximum at 400 nm (with an excitation maximum at 311 nm). It was found that the coupling reaction of 2-iodophenol leading to 2,2′-biphenol also showed outstanding selectivity in the formation of a particular isomer. This is manifested by the exceptional agreement of spectroscopic properties of the obtained product and those of the standard (solution of pure 2,2′-biphenol in MeOH). Both, the emission spectra (panels a and b of Figure 3) and the fluorescence decays (monoexponential, with the decay time of 3.2 ns; Figure 3c) recorded for the monoanion of the reaction product and the standard solution of 2,2′-biphenol monoanion are indistinguishable from each other. The reaction yield determined by comparing fluorescence intensities (Figure 3a; details are included in the Supporting Information) reaches 87% for the most optimized set of initial concentrations of those studied (entry 4 in Table 1).

**Figure 3:**
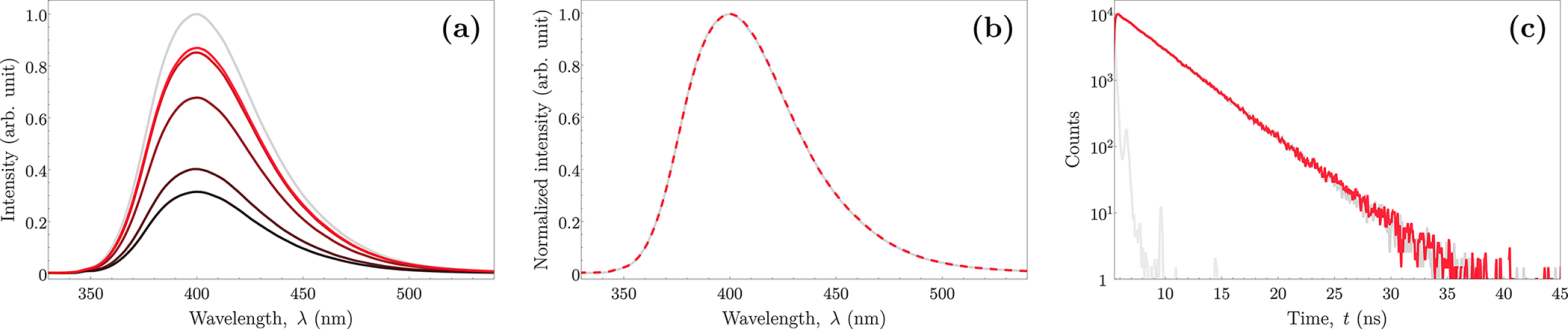
Emission spectra and fluorescence decays of the product obtained by the coupling of 2-iodophenol (red) and the reference solution of 2,2′-biphenol (gray) in alkalized MeOH. (a) Comparison of the fluorescence spectra of the 5 μM 2,2′-biphenol (standard) solution and the obtained product for each set of initial concentrations recorded with excitation at 311 nm; a lighter shade of red corresponds to a higher yield (4 > 3 > 1 > 2 > 5; Table 1). (b) Normalized emission spectra of the standard solution and the coupling product (for set 4). (c) Fluorescence decays in alkalized solutions recorded at 400 nm with excitation at 315 nm. The light gray color is the IRF.

Analysis of the data in Table 1 (yields as a function of initial concentrations) and derived metrics like turnover (discussed in the Supporting Information) suggest that the process under study is mediated by gold nanoparticles; however, it is not truly catalytic. The AuNPs undergo iodine coating (as shown in the TEM images; Figure 1), leading to their deactivation. This is expected as a result of the strong affinity of iodine for gold. Because existing literature does mention the potential for removing iodine coating from gold nanoparticles,^12^ the demonstrated activity of gold as a mediator under the such mild conditions holds fundamental significance and indicates that catalyst regeneration is an aspect worth exploring in future research.

As a final step of our research, to highlight the crucial role of gold in the investigated reaction, we also conducted the 4-iodophenol coupling reaction using different AuNPs synthesized through a citrate reduction route (i.e., without the use of PVP, of which the effect on the observed results was ruled out independently as part of the initial measurements; Figure S8 of the Supporting Information). Nanoparticles synthesized through the citrate reduction method are produced in an aqueous solution; therefore, it stands to reason that the reaction with them was carried out in water, thereby introducing a distinct set of conditions. Despite the notably larger size of these nanoparticles (10–15 nm) and water being a very poor solvent for the reactants, the reaction still achieved a yield of over 30% with high selectivity, as shown in Figure 4a. These results further prove the pivotal role of the gold metal in the reaction studied.

**Figure 4:**
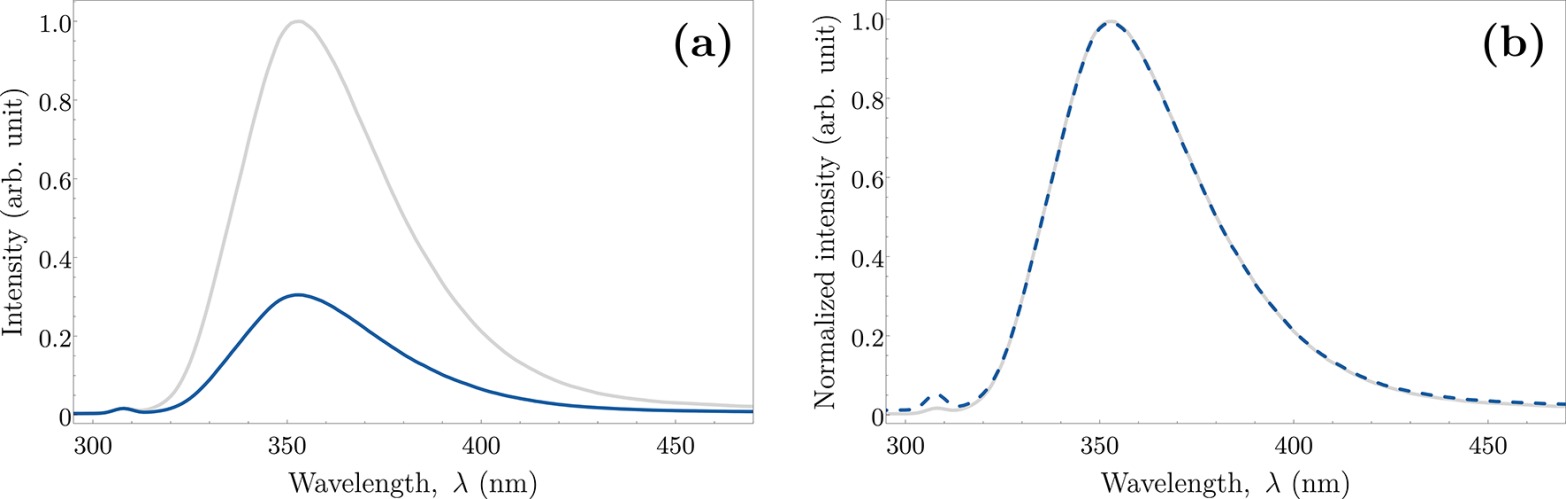
Emission spectra of the product obtained by the coupling of 4-iodophenol (navy blue) using AuNPs synthesized through a citrate reduction route and the reference solution of 4,4′-biphenol (gray) in water. (a) Comparison of the fluorescence spectra of the 5 μM 4,4′-biphenol (standard) aqueous solution and the obtained product recorded with excitation at 275 nm. (b) Normalized emission spectra of the standard solution and the coupling product. Fluorescence of unreacted 4-iodophenol is completely quenched in an aqueous solution.

The Ullmann homocoupling reaction, although undoubtedly associated with copper as a mediator in the past, has already been carried out successfully using gold;^13^ the required conditions, however, were characterized by a certain exorbitance. In the work of Karimi and Esfahani,^7^ gold nanoparticles were supported on bifunctional mesocanals of periodic mesoporous organosilicas (PMOs). The aryl iodide coupling reaction was carried out for 16 h in *N*-methylpyrrolidone, and the required temperature was 100 °C. Monopoli et al.^8^ showed that it is possible to carry out the aryl iodide coupling reaction also leading to high yields on gold nanoparticles obtained *in situ* under two different sets of conditions: in aqueous solution with TBAOH and glucose as a reductant and in molten TBAA in the presence of glucose, both at 90 °C with continuous stirring for 7 h. The work of Dhital et al.^9^ describes a remarkable homocoupling reaction of chloroarenes in the water/*N*,*N*-dimethylformamide (DMF) system at moderately low temperatures (27–45 °C), where bimetallic Au/Pd:PVP nanoparticles were used (highly significantly, monometallic Au:PVP showed no activity). However, a large addition of alkali was required for the reaction to occur, and the reaction was carried out under an argon atmosphere. In our case, the reaction was carried out at 21 °C, without stirring, in a common solvent, MeOH, without the addition of a base, using simple Au:PVP nanoparticles, obtaining surprisingly high yields for such mild conditions and an uncomplicated mediator. This, however, is not in contrast to previous reports; in fact, we hope it will act as a complement to them and provide a starting path for great improvement.

In summary, we have demonstrated that, as a result of a simple mixing of iodophenols with Au:PVP under room conditions without the addition of a base, the Ullmann homocoupling reaction occurs, where the observed emission spectra of resulting biphenols were previously erroneously attributed to the excimer formation of aryl iodides chemisorbed on gold nanoparticles.^6^ However, we firmly believe that the presented results not only offer an explanation of the observed phenomenon but are also valuable from the perspective of the chemistry of carbon–carbon coupling reactions. It should be noted that, despite the fact that the optimization of the process was beyond the scope of this work, surprisingly high reaction yields were achieved, which opens the path to its significant refinement starting from remarkably mild conditions using readily synthesizable AuNPs.

## Supporting Information Available

Experimental procedures, description of instrumental techniques, and details of calculations (PDF)
