# Supplementary material for: Deciphering Photoluminescence in an Aryl Iodides–Gold Nanoparticles System: Au-Mediated Homocoupling Reaction at a Low Temperature
Source: J Phys Chem Lett. 2024 Apr 4;15(14):3982–6. doi: 10.1021/acs.jpclett.4c00346 (PMC11017310; doi:10.1021/acs.jpclett.4c00346)
Supplement: Supplementary file 1 — jz4c00346_si_001.pdf [file jz4c00346_si_001.pdf]

Deciphering Photoluminescence in Aryl Iodides-Gold Nanoparticles System: Au-Mediated Homocoupling Reaction at Low Temperature

Paulina Rajchel-Mieldzioć and Piotr Fita*

Institute of Experimental Physics, Faculty of Physics, University of Warsaw

Pasteura 5, 02-093 Warsaw, Poland

E-mail: fita@fuw.edu.pl

Phone: +48 22 55 32 733

Synthesis and reaction

Reagents

All reagents were used as received without further purification. Hydrogen tetrachloroaurate trihydrate ($HAuCl_4 \cdot 3H_2O$, AmBeed), sodium tetraborohydride ($NaBH_4$, Sigma-Aldrich) and poly(N-vinylpyrrolidone) (PVP K-30, Sigma-Aldrich) were employed as precursors for the synthesis of metallic nanoparticles, while 4-iodophenol and 2-iodophenol (both from Sigma-Aldrich) were used as substrates in the coupling reaction. For comparative spectrophotometric measurements, standard solutions of 4,4'-biphenol and 2,2'-biphenol were used (obtained from Sigma-Aldrich and Thermo Scientific, respectively). Milli-Q grade water was used in the preparation of the nanoparticles, while all experiments were conducted with LC-MS grade methanol (J.T. Baker) as a solvent.

Synthesis of Au:PVP nanoparticles

PVP-stabilized AuNPs were synthesized based on the method described in the literature.[1] In summary, 555.5 mg of PVP (K-30) was added to an aqueous solution of $HAuCl_4$ (1 mM, 50 ml), and the mixture was stirred vigorously in a bath maintained at 0°C. After cooling, an aqueous solution of $NaBH_4$ (100 mM, 5 mL) was rapidly added, yielding a deep brown solution (Au:PVP, 1 at.%), which was subjected to further stirring for at least 30 min. 2 mL of the resulting solution was ultrafiltrated using a centrifugal filter unit (3 kDa cutoff), and the formed precipitate was washed with purified water. After repeated ultrafiltration, the resulting precipitate was lyophilized.

Additional synthesis of AuNPs for control experiments using Turkevich method

Trisodium citrate dihydrate (ACS reagent), purchased from Sigma-Aldrich, was used for the synthesis. To obtain nanoparticles with diameters small for the method used (10-15 nm), the process was based on information available in the literature.[2] A 50 mL aqueous solution of $HAuCl_4$ at a concentration of 0.25 mM was prepared and heated to a temperature exceeding 90°C. While stirring vigorously, 3.3 mL of preheated 30 mM citrate solution was added (resulting in a molar ratio of $HAuCl_4$ to citrate of approx. 1:7.9). The solution was heated and stirred for another 7 min, then removed from heating and cooled. The nanoparticles thus obtained were used for a control experiment, where their concentration (with respect to atomic gold) was brought to 100 μM.

Coupling reaction

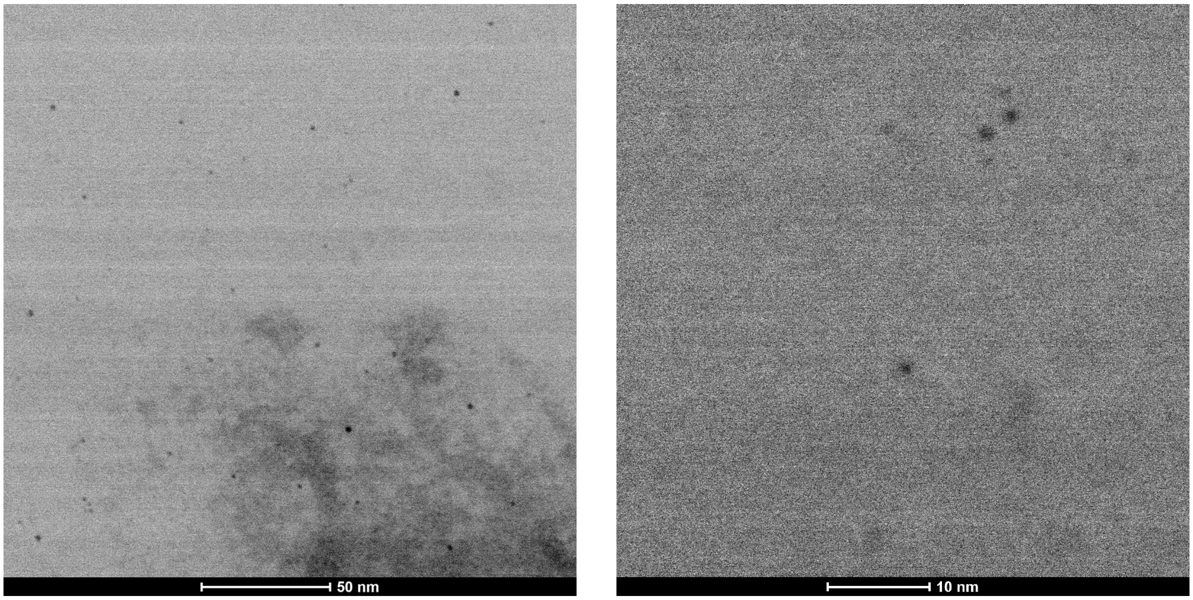

Figure S1: TEM images of Au:PVP dispersed in methanol

Upon lyophilization, the nanoparticles were quantitatively dissolved in 10 mL of methanol, yielding a solution with a gold concentration of 0.18 mM. TEM images of the obtained

Au:PVP in MeOH are shown in Fig. S1. In case of the sample **1** (Table 1), 1100 μL of the resulting stock solution of Au:PVP, 880 μL of methanol, and 20 μL of a 3 mM iodophenol solution were sequentially added into an Eppendorf tube. The resulting mixture (30 μM iodophenol) was left to incubate for 24 hours under room conditions (21°C, no stirring) before undergoing ultrafiltration (3 kDa cutoff) in order to separate the Au:PVP nanoparticles. Following this, the supernatant was diluted threefold and subjected to analysis for emission properties. The procedure for the remaining samples was analogous; the final concentrations were adjusted by selecting appropriate volumes of stock solutions and methanol. The dilution of the supernatant was also adjusted accordingly to obtain a solution for spectroscopic analysis with a limiting maximum product concentration (assuming 100% yield) of 5 μM.

Gold content in the supernatant

A research report of the analysis of the gold content of the supernatant (including technical details) is attached at the end of the Supporting Information. Codenames of the samples stand for: *para* – supernatant after 4-iodophenol coupling, *orto* – supernatant after 2-iodophenol coupling, respectively.

The concentration of gold ranged (depending on the sample) from 4.9 to 12.3 nM (nanomolar). This amount is over three orders of magnitude lower than the substrate concentration, which, considering the obtained yields, eliminates the possibility of a homogeneous mechanism for the studied reaction (where the primary argument is the depicted deposition of iodine on gold nanoparticles, Fig. 1 in the main text).

Instrumental techniques

Spectroscopic studies were carried out using the Horiba QuantaMaster 8075-11 spectrofluorometer equipped with the PPD850 photomultiplier (sensitivity in the range of 250-850 nm) and the DeltaTime kit for time-resolved measurements. Emission and excitation spec-

tra were recorded with sample solutions placed in 1x1 cm quartz cuvettes and excited by a built-in xenon lamp. Excitation and emission slits were set to 2 nm and the spectra were corrected for the sensitivity of the detector.

For measurements of fluorescence decays samples were excited with femtosecond pulses generated by frequency doubling (in a BBO crystal) output pulses of an optical parametric amplifier (Orpheus by Light Conversion) pumped by a 1030 nm femtosecond amplifier (Carbide by Light Conversion). The repetition rate of the pulses was set to 2 MHz. The excitation beam power was kept at the level ensuring no saturation and a linear response of the detector.

The instrument response function (IRF) was measured by scattering the excitation beam in a suspension of TiO_2 in water. Analysis of the fluorescence decays was performed using the Horiba FelixGX software by reconvolution of multiexpontial decays with the IRF and fitting the convoluted functions to the experimental decays.

HR TEM investigations were conducted on an FEI Talos F200X transmission microscope at 200 kV. The morphology and chemical composition were performed in TEM and STEM modes using high-angle annular dark-field imaging (HAADF). Energy-dispersive X-ray spectroscopy (Super-EDS by FEI) detector was used for mapping element distribution.

[1]NMR spectra were taken with a 600 MHz DDR2 spectrometer from Agilent.

Spectra

Spectroscopic analysis based on neutral form of 2,2'-biphenol

An analysis based on a comparison of the photophysical properties of the deprotonated form (monoanion) of 2,2'-biphenol provides the basis for identifying the 2-iodophenol coupling product (Fig. 3 in the main text). The same results are obtained when studies are based on the comparison of emission spectra and fluorescence decays of the neutral form of 2,2'-biphenol (Fig. S2).

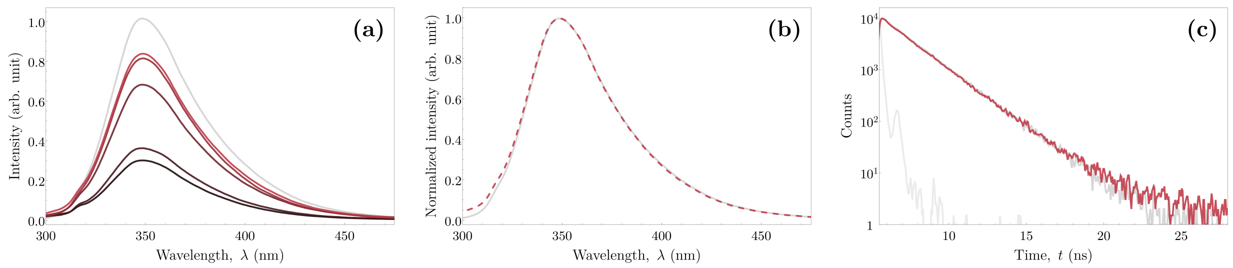

Figure S2: Emission spectra and fluorescence decays of the product obtained by the coupling of 2-iodophenol (burgundy) and the reference solution of 2,2'-biphenol (gray) in MeOH. **(a)** Comparison of the fluorescence spectra of the 5 μM 2,2'-biphenol (standard) solution and the obtained product for each set of initial concentrations recorded with excitation at 286 nm; lighter shade of burgundy corresponds to higher yield (**4** > **3** > **1** > **2** > **5**, Table 1). **(b)** Normalized emission spectra of the standard solution and the coupling product (for set **4**). **(c)** Fluorescence decays in neutral solutions recorded at 347 nm with excitation at 286 nm. Light gray color – IRF.

As in the case of the comparison of emission spectra and fluorescence decay of the alkalized coupling product of 2,2'-biphenol and the alkalized standard solution of 2,2'-biphenol (Fig. 3), we also observe exceptional agreement in the spectroscopic properties of the obtained product and the standard in the neutral solutions. This provides irrefutable confirmation of the identification of 2,2'-biphenol as a product of Au:PVP-mediated coupling of 2-iodophenol.

Spectroscopic analysis based on excitation spectra

The analysis presented in the main text, as well as in the previous part of the Supporting Information, is based on a comparison of the emission properties of biphenols obtained through the coupling of iodophenols and corresponding standard solutions. Additional validation of our conclusions is evident when examining the excitation spectra of the coupling reaction products alongside the excitation spectra of the corresponding standard solutions of biphenols (Figs. S3 – S5).

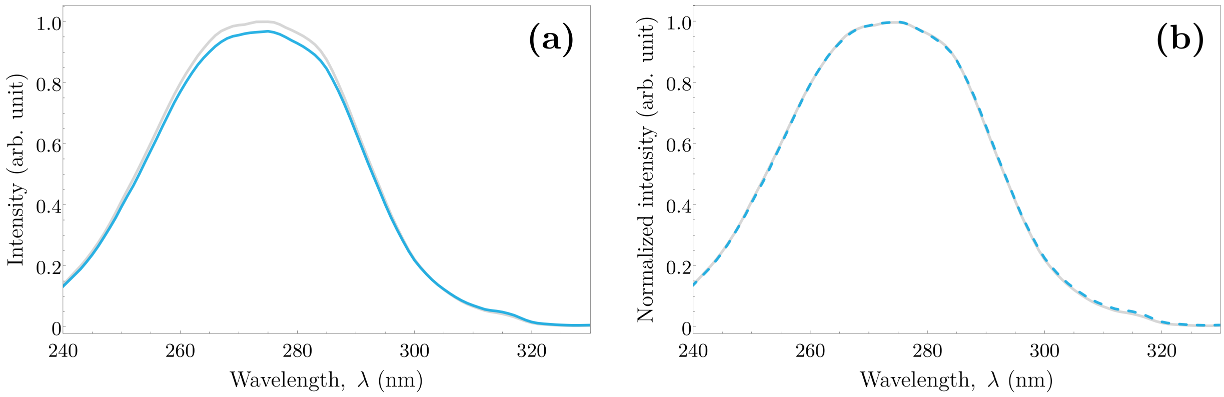

Figure S3: Excitation spectra of the product obtained by coupling of 4-iodophenol (blue) and reference solution of 4,4'-biphenol (gray). **(a)** Comparison of the excitation spectra of the 5 μM 4,4'-biphenol (standard) solution and the obtained product (for set **1**) recorded with emission at 353 nm. **(b)** Normalized excitation spectra of the standard solution and the coupling product (for set **1**).

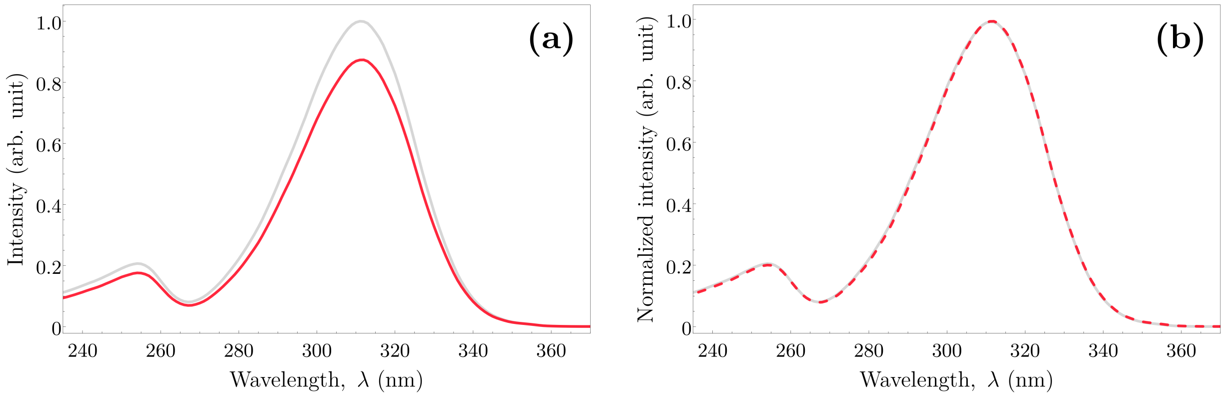

Figure S4: Excitation spectra of the product obtained by coupling of 2-iodophenol (red) and reference solution of 2,2'-biphenol (gray) in alkalized MeOH. **(a)** Comparison of the excitation spectra of the 5 μM 2,2'-biphenol (standard) solution and the obtained product (for set **4**) recorded with emission at 400 nm. **(b)** Normalized excitation spectra of the alkalized standard solution and the coupling product (for set **4**).

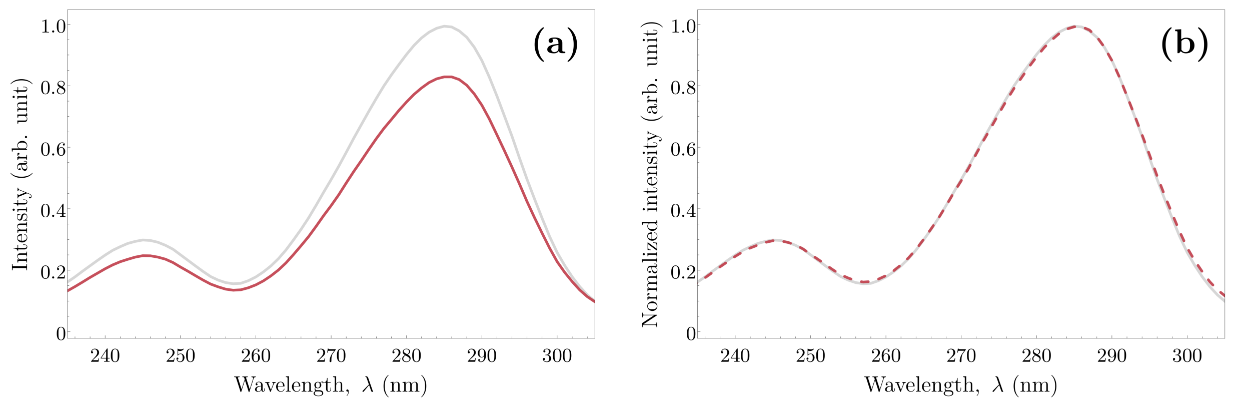

Figure S5: Excitation spectra of the product obtained by coupling of 2-iodophenol (burgundy) and reference solution of 2,2'-biphenol (gray) in neutral MeOH. **(a)** Comparison of the excitation spectra of the 5 μM 2,2'-biphenol (standard) solution and the obtained product (for set **4**) recorded with emission at 347 nm. **(b)** Normalized excitation spectra of the standard solution and the coupling product (for set **4**).

Spectra of standard solutions of 4,4'-biphenol and 2,2'-biphenol

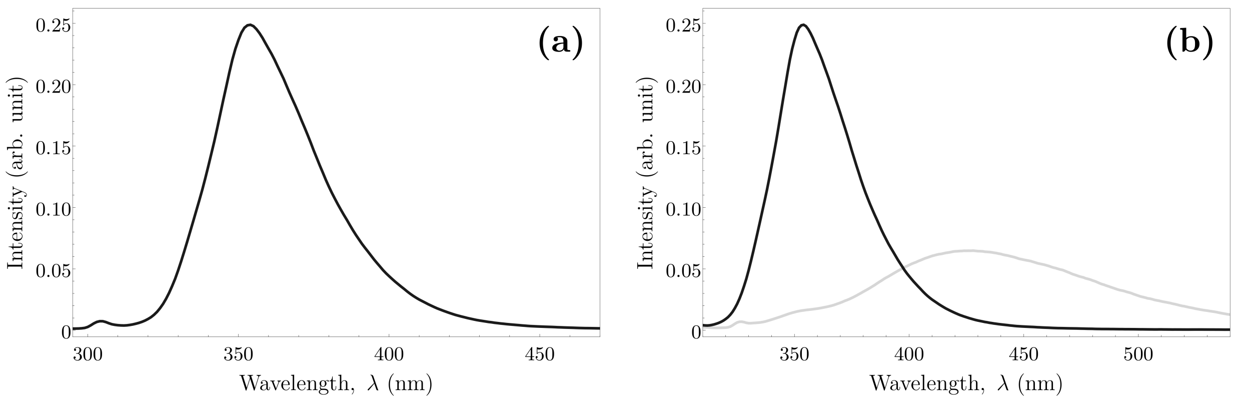

Figure S6: Emission spectra of the 5 μM standard solution of 4,4'-biphenol in methanol. **(a)** Spectrum of the neutral form of 4,4'-biphenol (black) recorded in the neutral solution with excitation at 275 nm. **(b)** Comparison of the spectrum of the neutral solution (excitation at 275 nm, black) and the spectrum of the deprotonated forms recorded in the alkalized solution with excitation at 293 nm (light gray).

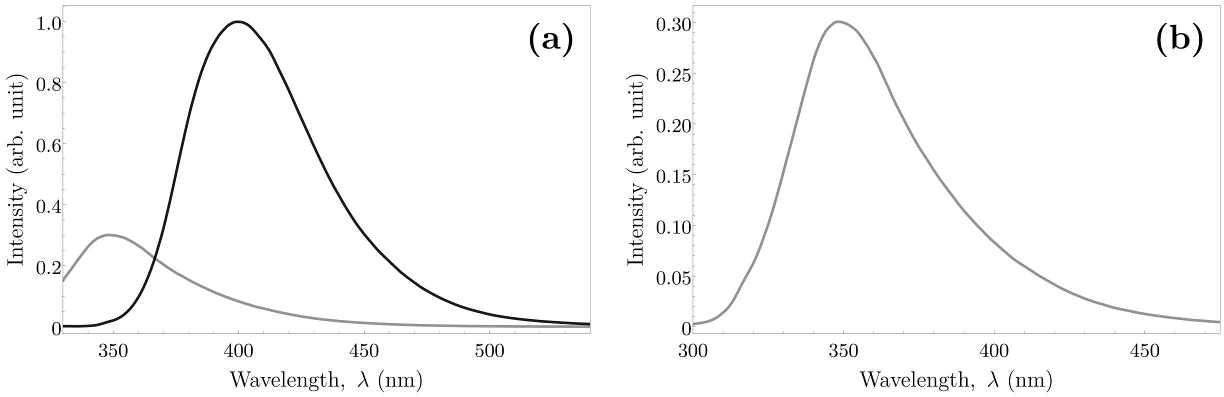

Figure S7: Emission spectra of the 5 μM standard solution of 2,2'-biphenol in methanol. **(a)** Spectra of deprotonated (monoanionic; black) and neutral (gray) forms of 2,2'-biphenol. Spectrum of the monoanion was recorded in the alkalized solution with excitation at 311 nm and the spectrum of the neutral form was recorded in the neutral solution with excitation at 286 nm. **(b)** For comparison: enlarged spectrum of the neutral form, excitation at 286 nm.

PVP spectra

The following is a study of polyvinylpyrrolidone (PVP) emission in methanol at a concentration corresponding to its hypothetical complete transition to the supernatant after the coupling reaction. It's an entirely impossible scenario, given the use of centrifugal filters with 3 kDa cut-off – but it serves to clearly demonstrate that PVP has no effect on the fluorescence of the products of the coupling reaction. The results are shown in Fig. S8. Diluting the solution caused a further decrease in the already residual signal – and the use of centrifugal filters equalized the signal with the level of the solvent.

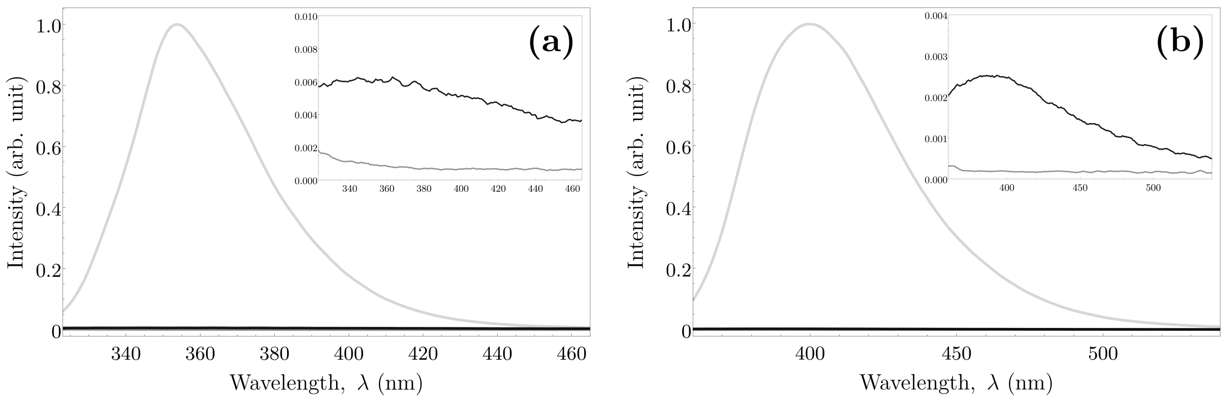

Figure S8: Emission spectra of the 5 μM standard solutions of biphenols and polyvinylpyrrolidone (PVP) in methanol. **(a)** Comparison of the emission spectra of the 5 μM 4,4'-biphenol (gray), pure methanol (dark gray) and PVP (black) recorded with excitation at 275 nm. Insert: Comparison of the emission spectra of pure methanol and PVP only. **(b)** Comparison of the emission spectra of the alkalized 5 μM 2,2'-biphenol (gray), pure alkalized methanol (dark gray) and alkalized PVP (black) recorded with excitation at 311 nm. Insert: Comparison of the emission spectra of pure methanol and PVP only (both alkalized).

^{1}H NMR spectra

Figs. S9 and S10 present ^{1}H NMR spectra confirming the presence of 4,4'-biphenol and 2,2'-biphenol, respectively, in the samples tested. What is worth noting, the amounts of the product were defined by the scale of the reaction, hence the small amount of tested products relative to the solvent. In addition, the evaporation process did not fully remove methanol, which is also present in the spectrum recorded in $CDCl_3$. However, this did not prevent the positive identification of the products.

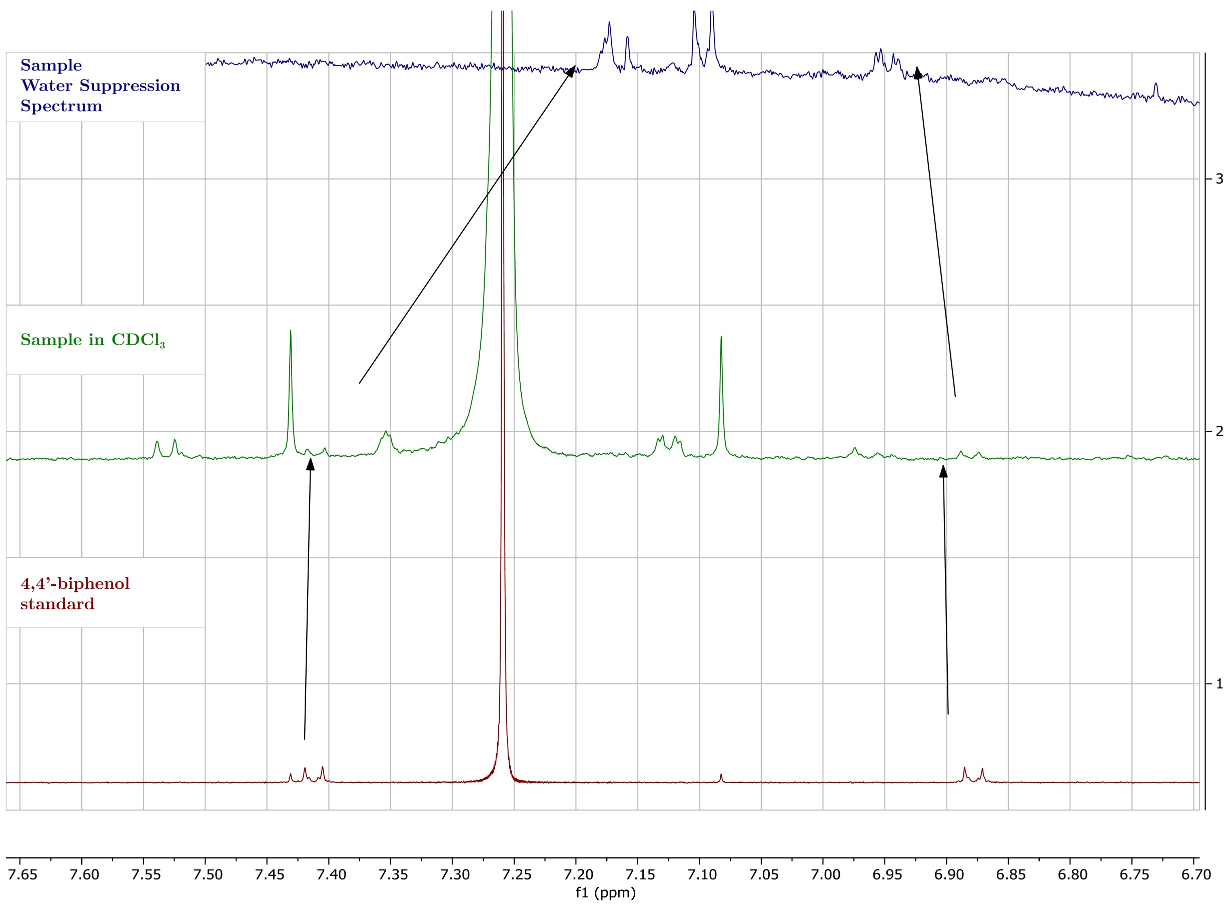

Figure S9: ^{1}H NMR spectra (aromatic region) of the product obtained by the coupling of 4-iodophenol (blue – spectrum in non-deuterated MeOH with added D_2O, green – spectrum in $CDCl_3$) and the reference solution of 4,4'-biphenol (red).

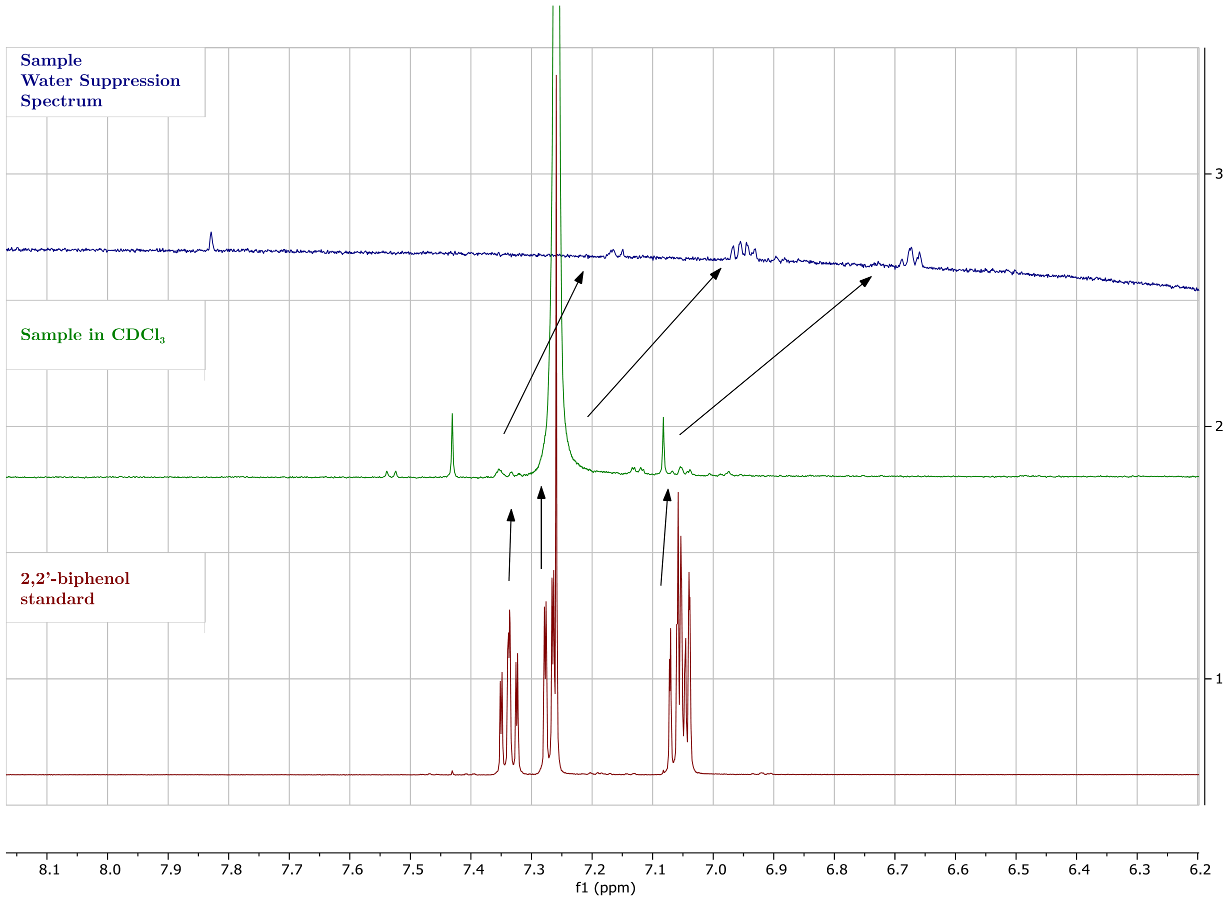

Figure S10: ^{1}H NMR spectra (aromatic region) of the product obtained by the coupling of 2-iodophenol (blue – spectrum in non-deuterated MeOH with added D_2O, green – spectrum in $CDCl_3$) and the reference solution of 2,2'-biphenol (red).

Estimation of reaction yield

The reaction yields of the syntheses were estimated by comparing the fluorescence intensity of a 5 μM standard solution of biphenol and appropriately diluted post-reaction solution, in which the maximum possible concentration of the product is also 5 μM. The concordance of the spectral shapes (Fig. 2b and Fig. 3b) and fluorescence decays (Fig. 2c and Fig. 3c) ruled out beyond any doubt the presence of other isomers than 4,4'-biphenol and 2,2'-biphenol, respectively – indicating the high selectivity of both reactions. For the coupling reaction of 4-iodophenol to 4,4'-biphenol, yield determination was based on the emission spectrum of

the neutral form of 4,4'-biphenol. In the case of the 2-iodophenol coupling to 2,2'-biphenol, yield assessment relied on the emission spectrum of its deprotonated (monoanion) form. The results obtained for each set of initial concentrations are shown in the Table S1 (Table 1 in the main text).

Table S1: Substrates, products and estimated yields of the Ullman homocupling reactions mediated by Au:PVP (1 at. %) in MeOH

Entry	Au:PVP[a] (μM)	iodophenol[b] (μM)	Yield (%) 4,4'-biphenol	Yield (%) 2,2'-biphenol
1	100	30	**97**	68
2	50	30	35	40
3	200	30	87	85
4	100	15	73	**87**
5	100	60	32	31

[a] Concentration of Au:PVP in relation to atomic gold

[b] Concentration of the substrate, 4-iodophenol or 2-iodophenol respectively

Au:PVP activity

Analysis of the changes in the reaction yield as a function of the initial substrate concentration (Table S1) allows us to conclude that the reaction is not truly catalytic, but follows a certain stoichiometry.

In an idealized case, one may consider that each surface atom is an active center in combination with one-to-one stoichiometry, i.e. that every surface gold atom corresponds to one iodine atom. Analysis based on such assumption is possible, with an appropriate determination of the number of gold atoms[3–5] in a nanoparticle of a given size and the total concentration of gold. However, this approach, while providing a theoretical reference value, overlooks several key factors: the potential inaccessibility of parts of the nanoparticle surface, the impact of quasi-permanent chemisorption of iodine on adjacent active sites – potentially altering the overall stoichiometry – and the fact that iodine introduction to gold nanoparticles results in their aggregation, as shown in Fig. 1 and also described in the literature.[6–8]

Importantly, if we assume that deviations have a similar impact across all samples, a

relative analysis becomes possible. This involves comparing the ratios of the calculated concentrations of the yielded products to the total gold concentration in each data set with one another. Such approach is based on the fact that, assuming a homogeneous population of nanoparticles, the total concentration of gold is directly proportional to their number, and thus to the active area – regardless of any variations.

Table S2: Ratios of the calculated concentrations of the yielded products to the total gold concentration

Entry	$\frac{\text{4,4'-biphenol}^a}{\text{Au:PVP}}$	$\frac{\text{2,2'-biphenol}^a}{\text{Au:PVP}}$
1	**1**	**0.70**
2	0.73	0.83
3	*0.45*	*0.44*
4	*0.37*	*0.45*
5	0.67	0.65

[a] Ratio of concentrations in arbitrary units, normalized to the value for 4,4'-biphenol obtained for the highest reaction yield

The results of the calculations are shown in the Table S2, where the typeface (bold, italic, normal) refers to the same concentration ratio of the substrate (iodophenol) and gold. Several observations can be made by comparing the reaction yields (Tab. S1) and the relative concentration ratios (Tab. S2). First, a reduction of the number of nanoparticles with respect to optimal conditions leads to the significant decrease of the yield (from 85-97% down to 30-40%). This loss of the yield occurs when the concentration of the nanoparticles drops down below twice the concentration of the substrate. Since for nanoparticles of the size used in the study roughly half of the atoms reside on their surface one can conclude that the limit of the yield is reached when majority of active sites are occupied with iodine. On the other hand, the yield decreases also for an excess of nanoparticles. One can interpret it in such a way that for an excess of active sites substrate molecules are adsorbed at too distant sites to allow the homocoupling reaction and the latter occurs only between molecules adsorbed at appropriate relative positions. This is reflected by the concentration ratios: For sets **3** and

4 the ratios are lower than for sets **2** and **5**, even though the reactions yields are much lower for the latter sets.

Altogether the results indicate that the homocoupling reaction is stoichiometric with respect to active sites at gold nanoparticles, however its potential catalytic character was shown by reusing nanoparticles used in a reaction. The nanoparticles used in the set **1** were centrifuged and reused under the same conditions in the 4-iodophenol coupling reaction. As shown in Fig. S11, the yield obtained was 42% (compared to 97% in the first cycle). This results in a total yield of 69%, calculated with respect to the total substrate concentration used in both reactions. In comparison, in the corresponding experiment (set **5**) with the same concentration of nanoparticles and a substrate concentration of 60 μM (as opposed to the subsequent double introduction of the same amount of the substrate at a concentration of 30 μM), significantly lower yield of 32% was achieved. This implies that merely centrifuging the post-reaction nanoparticles, without an explicit focus on purification, leads to a partial cleaning of their surface. Consequently, this outcome also introduces the potential for their reuse, especially if the are deliberately cleaned between consecutive reactions.

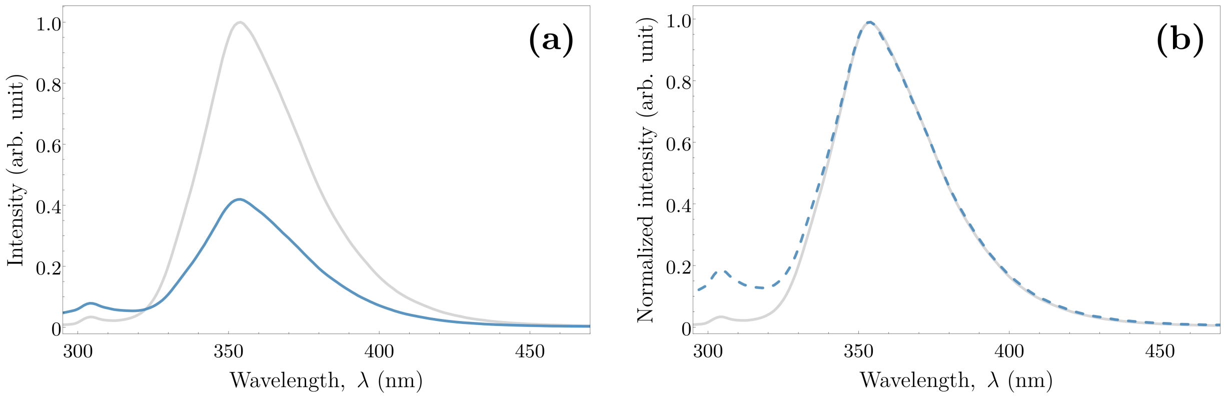

Figure S11: Emission spectra of the product obtained by the coupling of 4-iodophenol (steel blue) utilizing previously used Au:PVP and of the reference solution of 4,4'-biphenol (gray) in MeOH. **(a)** Comparison of the fluorescence spectra of the 5 μM 4,4'-biphenol (standard) solution and the obtained product recorded with excitation at 275 nm. **(b)** Normalized emission spectra of the standard solution and the coupling product. The short-wavelength edge of the spectrum of the post-reaction solution shows a contribution from the unreacted 4-iodophenol.

References

(1) Tsunoyama, H.; Sakurai, H.; Ichikuni, N.; Negishi, Y.; Tsukuda, T. Colloidal Gold Nanoparticles as Catalyst for Carbon-Carbon Bond Formation: Application to Aerobic Homocoupling of Phenylboronic Acid in Water. *Langmuir* **2004**, *20*, 11293–11296.

(2) Dong, J.; Carpinone, P. L.; Pyrgiotakis, G.; Demokritou, P.; Moudgil, B. M. Synthesis of Precision Gold Nanoparticles Using Turkevich Method. *KONA Powder Part. J.* **2020**, *37*, 224–232.

(3) Lu, Y.; Wang, L.; Chen, D.; Wang, G. Determination of the Concentration and the Average Number of Gold Atoms in a Gold Nanoparticle by Osmotic Pressure. *Langmuir* **2012**, *28*, 9282–9287.

(4) Ackerson, C. J.; Jadzinsky, P. D.; Sexton, J. Z.; Bushnell, D. A.; Kornberg, R. D. Synthesis and Bioconjugation of 2 and 3 nm-Diameter Gold Nanoparticles. *Bioconjugate Chem.* **2010**, *21*, 214–218.

(5) Mori, T.; Hegmann, T. Determining the Composition of Gold Nanoparticles: a Compilation of Shapes, Sizes, and Calculations Using Geometric Considerations. *J. Nanoparticle Res.* **2016**, *18*, No. 295.

(6) Cheng, W.; Dong, S.; Wang, E. Iodine-Induced Gold-Nanoparticle Fusion/Fragmentation/Aggregation and Iodine-Linked Nanostructured Assemblies on a Glass Substrate. *Angew. Chem. Int. Ed.* **2003**, *42*, 449–452.

(7) Singh, S.; Pasricha, R.; Bhatta, U. M.; Satyam, P. V.; Sastry, M.; Prasad, B. L. V. Effect of Halogen Addition to Monolayer Protected Gold Nanoparticles. *J. Mater. Chem.* **2007**, *17*, 1614–1619.

(8) Wang, J.; Li, Y. F.; Huang, C. Z. Identification of Iodine-Induced Morphological Transformation of Gold Nanorods. *J. Phys. Chem. C* **2008**, *112*, 11691–11695.

UNIWERSYTET WARSZAWSKI

Centrum Nauk Biologiczno-Chemicznych Uniwersytetu Warszawskiego

The University of Warsaw
Biological and Chemical Research Centre
ul. Żwirki i Wigury 101;
02-089 Warszawa

Warsaw, 07.12.2023

Research report:

Analysis of gold content.

The purpose of the analysis was to determine content and possible determination of trace amounts of gold in samples provided by the client:

Sample Para
Sample Orto

The method was developed and optimized.

In order to estimate the gold level in the samples provided by the client, the ICP MS (Inductively Coupled Plasma Mass Spectrometry) technique was used. As the solvent was methanol, it was necessary to use a desolvation nebulizer (Aridus 3, Cetac). Due to significant memory effects associated with introducing gold in unknown form into the spectrometer, analytical signals from the samples were first recorded. Then, signals from gold standards in methanol were recorded. The concentration of the standards was increased until the analytical signal from the standard and the sample was the same.

The samples and standards were shaken for 1 minute immediately before measurement. Speciation form of Au in the solutions remains unknown as the ICP MS technique used in this mode is not sensitive to the chemical form of Au. The table below shows the results:

Table 1. Gold content in samples obtained using the ICP MS technique [μg/kg].

Sample	Results with uncertainty [μg/kg]
**Sample Para**	1.22 μg/kg, U(n=5, k=2) = 0.13
**Sample Orto**	3.07 μg/kg, U(n=7, k=2) = 0.30

Results prepared:

Karasiński
